# Takotsubo Cardiomyopathy Presenting as Wellens’ Syndrome

**DOI:** 10.5811/cpcem.2017.1.32297

**Published:** 2017-05-24

**Authors:** R. Scott Taylor, Leif Skjerli, John Ashurst

**Affiliations:** * Duke LifePoint Conemaugh Memorial Medical Center, Department of Emergency Medicine, Johnstown, Pennsylvania; † Lake Erie College of Osteopathic Medicine, Erie, Pennsylvania

## Abstract

Takotsubo cardiomyopathy, also known as apical ballooning syndrome and stress cardiomyopathy, is a transient systolic and diastolic left ventricular dysfunction with a variety of cardiac wall-motion abnormalities that is increasingly being associated with significant morbidity and mortality. Wellens’ syndrome is an electrocardiographic (ECG) pattern in a pain-free patient that is indicative of critical occlusion of the left anterior descending coronary artery requiring immediate cardiac catheterization. The authors report a case of a patient presenting with ECG findings consistent with Wellens’ syndrome that was later found to have Takotsubo cardiomyopathy with angiographically normal coronary arteries on cardiac catheterization after a seizure.

## INTRODUCTION

Electrocardiographic (ECG) recognition of ST-elevation myocardial infarction (STEMI) patterns decreases patient morbidity and mortality and is an essential skill for emergency physicians.^1^ In addition, there has been increasing evidence, as well as social media blogs, stressing the importance of recognizing STEMI equivalent patterns on ECG. Wellens’ syndrome is identified by a specific ECG pattern without complaint of chest pain and is typically indicative of critical occlusion of the left anterior descending coronary artery (LAD). Due to this, Wellens’ syndrome is commonly referred to as a STEMI equivalent.^2^ Alternatively, there are ECG findings that can appear to be a STEMI, but are rather STEMI mimics. One such instance is that of Takotsubo cardiomyopathy.^2^ Takotsubo cardiomyopathy is commonly associated with elderly women experiencing an intense physical or emotional event. In this disease apical ballooning occurs, which causes ST segment changes on ECG consistent with STEMI. The authors report a case of a patient presenting with ECG findings consistent with Wellens’ syndrome who was later found to have Takotsubo cardiomyopathy.

## CASE REPORT

A 65-year-old female with a history of a seizure disorder was brought to the emergency department (ED) by ambulance after experiencing three seizures earlier in the day. Emergency medical services personnel were present for the third seizure and treated her with diazepam 5 mg intravenously prior to arrival to the hospital. Her family member stated that she had not taken any of her daily medications due to a two-day history of nausea, vomiting and diarrhea. On initial evaluation in the ED, the patient denied chest pain, dyspnea, or other complaints.

In addition to the seizure disorder, her medical history was significant for peripheral artery disease, obstructive sleep apnea, hypothyroidism, and migraines. Her home medications included clopidogrel, carbamazepine, lamotrigine, topiramate, and levothyroxine. She had denied current use of tobacco, alcohol, or illicit drugs.

Physical exam revealed that the patient had a fluctuating level of consciousness, consistent with a postictal state, but no lateralizing neurological deficits. Her vital signs were a temperature of 36.3 degrees Celsius, pulse of 118 beats per minute, respiratory rate of 20 breaths per minute, blood pressure of 146/69 mmHg, and an oxygen saturation of 96% on three liters of supplemental oxygen by nasal cannula. Her cardiovascular exam was only remarkable for a regular tachycardia. The remainder of the physical exam was unremarkable.

Chest radiograph and computed tomography of the head were unremarkable. An electrocardiogram upon arrival revealed sinus tachycardia with rate of 114 beats per minute with biphasic T-waves in leads V2–V4 with a noted 1 mm ST-elevation in V1 and a <1 mm ST-elevation in aVL (Image). Remarkable labs included a white blood count of 16 × 10^3^ mm,^3^ Troponin I of 2.42 ng/mL, CK-MB of 18.5 ng/mL, and a lactate of 3.0 mmol/L. Urinalysis was incidentally consistent with a urinary tract infection.

Interventional cardiology was consulted and it was determined that the patient would require urgent cardiac catheterization. In addition to her home dose clopidogrel, the patient was given aspirin 324 mg orally, metoprolol 5 mg IV and was given a weight-based dose of heparin. The patient was then taken for cardiac catheterization, which revealed angiographically normal coronary arteries, moderately impaired left ventricular function with ejection fraction (EF) of 35%, and wall-motion abnormalities of akinesis of the apical and apical septal wall, as well as severe hypokinesis of the anterolateral, diaphragmatic, and inferolateral wall. Interventional cardiology described the wall-motion abnormalities as being consistent with Takotsubo cardiomyopathy. Post-cardiac catheterization echocardiography revealed an EF of 30–35%, apical akinesis, severe hypokinesis of distal anteroseptal, anterior, lateral, and inferior walls, with compensatory basal hyperkinesis. She was started on a beta-blocker as well as an ACE inhibitor with recommended follow-up echocardiogram in three months to evaluate for improvement.

CPC-EM Capsule

What do we already know about this clinical entity?

Wellens’ syndrome is a STEMI equivalent that requires prompt interventional cardiology consultation. However, it is rarely associated with Takotsubo cardiomyopathy.

What makes this presentation of disease reportable?

Although both Wellens’ syndrome and Takotsubo cardiomyopathy have been reported in the literature, rarely are they seen in the same patient encounter.

What is the major learning point?

The emergency physician should keep a broad differential when faced with either a STEMI mimic or an equivalent.

How might this improve emergency medicine practice?

As more cases are reported, further studies may show an association between STEMI mimics and STEMI equivalents.

## DISCUSSION

Takotsubo cardiomyopathy, also known as apical ballooning syndrome or stress cardiomyopathy, is an acute-onset regional left ventricular dysfunction that is associated with chest pain and heart failure. It is becoming an increasingly recognized heart disease, affecting 5.2/100,000 women and 0.6/100,000 men in the United States.^3^ The disease occurs most frequently in women greater than 55 years, although all ages and both sexes can be affected.^3^ Although it is commonly preceded by an intense physical or emotional triggering event, such events are not identified in approximately one third of patients.^4^,^5^ Affected patients typically present with chest pain, dyspnea, and transient ST-segment elevation, which mirrors the presentation of an acute myocardial infarction (MI).^4^,^6^,^7^ Troponin I levels are usually elevated in this disease, but to a lesser extent than in a MI (1.8x vs 6x).^4^ Diagnostic criteria were first proposed by the Mayo Clinic, and subsequently modified by Prasad et al. in 2008 (Table).^5^

As Takotsubo cardiomyopathy presents similarly to acute coronary artery occlusion, it is usually diagnosed by cardiac catheterization that shows normal coronary arteries and wall-motion abnormalities.^6^ The cardiomyopathy is usually self-limited, and with supportive treatment, rapid return to normal cardiac function can be expected.^8^ Although self limiting, it is associated with ventricular tachycardia, ventricular thrombus, and ventricular rupture, which may increase the diseases morbidity and mortality.^6^

In our case, the patient presented with an ECG and clinical findings consistent with Wellens’ syndrome but was found to have Takotsubo cardiomyopathy on cardiac catheterization. It is unusual for a STEMI mimic to manifest as a STEMI-equivalent finding on ECG. This case highlights the importance of considering a broader differential diagnosis when confronted with ECG findings suspected to be STEMI equivalents. However, there will still be a need for invasive cardiac testing in consultation with cardiology to rule out MI.

Interestingly, it was also hypothesized that the patient’s triggering event may have been caused by seizure activity. In 2011, Stöllberger et al. reported several cases of Takotsubo cardiomyopathy triggered by seizure activity.^12^ None of these cases were reported, or suspected, to have a Wellens’ syndrome. Most of the patients in the case-series experience did, however, experience chest pain and/or hemodynamic deterioration, which was not present in our case. Wellens’ syndrome is typically not preceded by seizure activity as the underlying etiology is a coronary lesion. No association of Wellens’ syndrome with seizure activity could be found.

## CONCLUSION

Wellens’ syndrome, originally described in 1982, is a characteristic ECG finding that signifies critical LAD stenosis; however, the patient is usually pain-free at the point the ECG is obtained.^9^,^10^ It is important to recognize this syndrome to ensure timely transfer to the cardiac catheterization suite, as well as to avoid any testing that could induce MI, especially provocative stress imaging.^11^ There are two types of ECG findings associated with this syndrome. Type A (25% of cases) is identified by biphasic T-waves in V2–3, while type B’s findings are deeply inverted T-waves in V2–3.^11^

## Figures and Tables

**Image:**
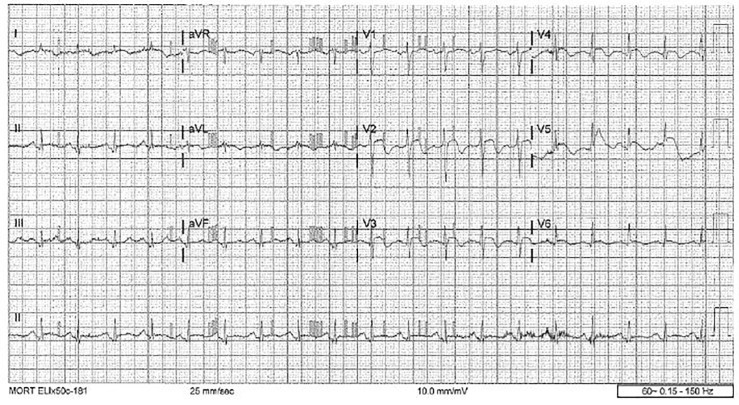
Electrocardiogram depicting Wellens’ syndrome.

**Table:** Diagnostic criteria for the diagnosis of Takotsubo cardiomyopathy.^5^

1.	Transient hypokinesis, akinesis, or dyskinesis of the left ventricular mid segments with or without apical involvement; the regional wall motion abnormalities extend beyond a single epicardial vascular distribution; a stressful trigger is often, but not always, present.
2.	Absence of obstructive coronary disease or angiographic evidence of acute plaque rupture.
3.	New ECG abnormalities (either ST-segment elevation and/or T-wave inversion) or modest elevation in cardiac troponin.
4.	Absence of pheochromocytoma or myocarditis.

*ECG*, electrocardiogram
